# Increase in the risk of snakebites incidence due to changes in humidity levels: A time series study in four municipalities of the state of Rondônia

**DOI:** 10.1590/0037-8682-0377-2019

**Published:** 2020-02-21

**Authors:** Alex Augusto Ferreira e Ferreira, Valdison Pereira dos Reis, Charles Nunes Boeno, Jaina Rodrigues Evangelista, Hallison Mota Santana, Suzanne Nery Serrath, Jéssica Amaral Lopes, Cristina Matiele Alves Rego, Maria Naiara Macedo Tavares, Mauro Valentino Paloschi, Neriane Monteiro Nery, Alessandra da Silva Dantas, Moreno Magalhães S. Rodrigues, Juliana Pavan Zuliani

**Affiliations:** 1 Fundação Oswaldo Cruz, Laboratório de Imunologia Celular Aplicada à Saúde, Porto Velho, RO, Brasil.; 2 Fundação Oswaldo Cruz, Laboratório de Análise e Visualização de Dados, Porto Velho, RO, Brasil.; 3 Agência Estadual de Vigilância em Saúde, Coordenação Estadual de Acidentes por Animais Peçonhentos, Porto Velho, RO, Brasil.; 4 Universidade Federal de Rondônia, Departamento de Medicina, Porto Velho, RO, Brasil.

**Keywords:** Epidemiology, Snakebites, Climatic variables, Bayesian Modelling

## Abstract

**INTRODUCTION:**

Snakebites represent a serious global public health problem, especially in tropical countries. In Brazil, the incidence of snakebites ranges from 19 to 22 thousand cases per 100000 persons annually. The state of Rondônia, in particular, has had an increasing incidence of snakebites.

**METHODS:**

A retrospective cross-sectional study on snakebites was conducted from January 2007 to December 2018. Brazil’s Information System for Notifiable Diseases was queried for all snakebites reported in Porto Velho, Ariquemes, Cacoal, and Vilhena. Data on land surface temperatures during the day and night, precipitation, and humidity were obtained using the Google Earth Engine. A Bayesian time series model was constructed to describe the pattern of snakebites and their relationship with climate data.

**RESULTS:**

In total, 6326 snakebites were reported in Rondônia. Accidents were commonly caused by *Bothrops* sp. (n=2171, 81.80%). Snakebites most frequently occurred in rural areas (n=2271, 85.5%). Men, with a median age of 34 years (n=2101, 79.1%), were the most frequent bitten. Moderate clinical manifestation was the most common outcome of an accident (n=1101, 41.50%). There were clear seasonal patterns with respect to rainfall, humidity, and temperature. Rainfall and land surface temperature during the day or night did not increase the risk of snakebites in any city; however, changes in humidity increased the risk of snakebites in all cities.

**CONCLUSION:**

This study identified the population exposed to snakes and the influence of anthropic and climatic factors on the incidence of snakebites. According to climate data, changes in humidity increased the risk of snakebites.

## INTRODUCTION

Snakebites represent a serious global public health problem, especially in tropical countries^1^. Globally, approximately 2.1 million cases of snakebites are reported

per year, with an average mortality ranging from 81000 to 138000 cases^2^. Approximately 300000 persons have been reported to survive snakebite accidents, but with permanent disability or disfigurement^1^. Considering these worrisome statistics, in June 2017, the World Health Organization (WHO) decided to re-include snakebites on the list of neglected tropical diseases and classified it under category A diseases^3^.

In Brazil, the incidence of snakebites ranges from 19 to 22 thousand cases annually^4^. With respect to geographic regions, the center-west region experiences 33 snakebites/100000 inhabitants, and thus, records the highest rate, followed by the north (24 snakebites/100000 inhabitants), south (16 snakebites/100000 inhabitants), south-east (13 snakebites/100000 inhabitants) and north-east regions (07 snakebites/100000 inhabitants)^4^.

Most of these envenomings have been caused by the snakes in the *Bothrops* and *Crotalus* genera, followed by the *Lachesis* and *Micrurus* genera. Approximately 70 species of venomous snakes are found in the broad Brazilian territory^5^. 

The clinical manifestations of envenomations include coagulation disorders; cardiac, muscular, and renal lesions; and sepsis. These clinical aspects can be categorized as mild, moderate, or serious, depending on various factors, including the snake species, amount of inoculated venom, physiological conditions, and age of the victim^5^. The treatment for snakebites is based on a precise diagnosis and application of a specific antiserum, according to the signs and symptoms of the victim.

It has been noted that the occurrence of snakebites is associated with climatic, environmental, and socioeconomic factors, such as an increase in anthropogenic activity, mainly in field work^6^. Rondônia is located in the Amazon region, and owing to intense agricultural activity, the state stands out for having an increasing number of snakebite incidents^7^. Another important factor that increases the risk of snakebites is deforestation, associated with an increase in forest fires^8^ and population growth, mainly caused by the recent construction of the hydroelectric power plants of Santo Antônio and Jirau^9^.

Thus, considering the need to recommend public health policies to improve the awareness and prevention of snakebites in municipalities with the greatest risk of incidence, we performed a survey of the reported snakebites in Rondônia from 2007 to 2018, with the following objectives: (I) to analyze the pattern of snakebites in the municipalities of Porto Velho, Ariquemes, Cacoal, and Vilhena; (II) to evaluate the relationship between the occurrence of snakebites and environmental factors in the municipalities under study; and (III) to evaluate the seasonality of snakebites.

## METHODS

### Study design and database

We conducted a retrospective cross-sectional study on snakebites reported in Rondônia State, using data from the Information System for Notifiable Diseases (SINAN), from January 2007 to December 2018. These data were made available for us by Agência Estadual de Vigilância em Saúde. SINAN included data about patients who were afflicted by a venomous animal and assisted by any health institution (public or private). The database was queried for all cases of snakebite accidents reported in Porto Velho, Ariquemes, Cacoal, or Vilhena; the clinical and social aspects of the accidents were retrieved. A python script with details about the query, variable selection, and transformation is available at https://github.com/rodriguesmsb/snakesProject/scripts. Furthermore, in the same repository, scripts used for data analyses, supplementary figures, and tables could be found.

### Climatic data sources

Data regarding the land surface temperature (LST) during the day and night, precipitation, and humidity were obtained from the Google Earth Engine (https://code.earthengine.google.com/assessed on June 24, 2019). The Moderate-Resolution Imaging Spectroradiometer (MODIS) was used to obtain the LST. This product provides an average 8-day LST with a resolution of 1000 m^10^. Two bands (i.e., LST_Day_1 km and LST_Night_1 km) were used to extract the day (LSTD) and night (LSTN) temperatures, respectively. The monthly precipitation data were obtained using information from the TRMM 3B43 dataset. TRMM 3B43 merges microwave data from multiple satellites to estimate daily precipitation with a resolution of 0.25 arc degrees^11^. Humidity data were obtained from Global Land Data Assimilation System (GLDAS). GLDAS generates information on a series of land surface states and fluxes through data modeling and assimilation techniques^12^.

Demographic data were obtained from the Brazilian Institute of Geography and Statistics (IBGE). IBGE provided files with population counts and estimates for each municipality, since 1992. Data were downloaded from a DATASUS repository (http://www2.datasus.gov.br).

### Statistical Analysis

Descriptive analyses were conducted to describe the main aspects of the snakebite cases. Point estimates of variables, such as sex, accident severity, and age, were computed according to their nature (i.e., proportions for categorical data and means or median for numerical data).

Temporal aspects of climate data and snakebite incidence per 100,000 inhabitants in each city were explored by plotting monthly levels during the study period. Overall features of the data, as trend and seasonality, were obtained using visualization techniques.

A Bayesian time series model was constructed to describe the pattern of snakebite cases in each city and their relationship with climate data. This model can be expressed in a general form as:

$$\gamma_{t}\;\sim \;Poisson\;\left(\mu_{t}\right)=Poisson\left(e_{t}p_{t}\right)$$

$$log\mu_{t}=log\;e_{t}+\;\alpha +\;B_{1}\;rainfall_{t}+B_{2}\;LTSD_{t}+B_{3}+\;LTSD_{t}+\;\omega_{t}$$

Where ω_t_ is a hyperparameter specifying temporal correlation. In this case, given the ordered vector, ω_1_,...,ω_t_, a random walk (rw) model of order 1 was defined. This implies that ω_t_ depends on the previous t-1 elements. Thus, the conditional distribution of ω_t_ is:

$${\omega /\omega }_{t}-1\;\sim \;N(\omega_{t}-1,\;\alpha^{2})$$

Non-informative prior distributions were used for each β (i.e., β ~ N(0,1000). Diffuse logGamma distributions on the logarithm were used for precisions related to the hyperparameters.

The model described above was fitted using the Integrate Nested Laplace Approximation (INLA) technique. INLA is an approximate method for Bayesian inference of latent Gaussian models^13^. All analyses were conducted in the free statistical software, R (R Development Core Team, 2019)^14^ using mainly the functions present on tidyverse^15^ and INLA^16^. R scripts (HelpFunctions.R and S2B Analysis.Rmd) as well supplementary figures are provides trough a Github repository avaliable at: https://github.com/rodriguesmsb/snakesProject.

RESULTS

### General description of snakebites cases

In total, 6326 snakebite cases were recorded in Rondônia from January 2007 to December 2018. Less than 5% (n=332) of the reported accidents involved nonvenomous species (e.g., snakebite caused by *Boa constrictor*), and data on these accidents were excluded for further analyses. Approximately 42% (n=2655) of the reported cases occurred in the studied area.

Snakebite accidents were commonly caused by *Bothrops* sp. (n=2171, 81.80%), followed by *Laquesis* sp. (n=80, 3.1%), *Micrurus* sp. (n=35, 1.30%), and *Crotalus* sp. (n=32, 1.21%). Rural areas were the most common places for the snakebites to occur, with 2271 (85.5%) cases. Mainly, men, with a median age of 34 years (n=2101, 79.1%), were the victims. The snakebites most often occurred on the feet, as with 1343 (50.6%) cases, or other parts of the inferior extremities, such as the legs (n=671, 25.3%) or toes (n=230, 8.6%). 

The evaluation of snakebite severity in Brazil is standardized according to criteria established by the Ministry of Health (MH) through the diagnostic manual and treatment of envenomations. They can be classified as mild, moderate, or severe. There were 992 (37.4%) mild cases and 476 (18%) severe cases of the 2655 total snakebite cases. Thus, the most common outcome of a snakebite was moderate (n=1101, 41.50%) in severity. Of the 2655 victims, 2515 (approximately 95%) survived. During the whole study period, only two victims (<1%) died as a result of the snakebites, demonstrating the low lethality. 

### Temporal pattern of variables

**FIGURE 1:**
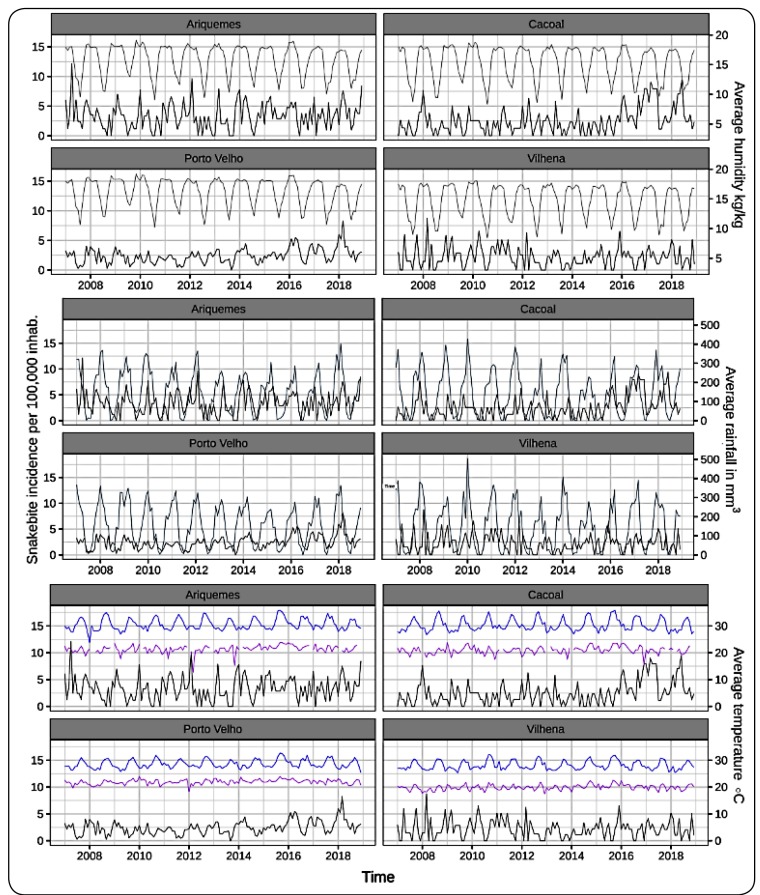
Time series of snakebite incidence and climatic variables in four cities of Rondônia, Brazil (2007-2018). The blue and purple lines are the series of the average maximum and minimum temperatures, respectively.

The overall monthly incidence of snakebites in the Porto Velho municipality was 2.41 (95% credible interval [CrI]: 2.21-2.62) cases per 100000 inhabitants. Here, September was the month with the lowest incidence. In this city, the first five months of each year had similar snakebite incidences (Figure 1). Further, the lowest incidence was observed in 2011, with 83 snakebite accidents (per 100000 inhabitants) notified in this city. The highest incidence (45.20 cases per 100,000 inhabitants) was observed in 2018. In the Cacoal municipality, the annual incidence of snakebites was the lowest in 2007 (18.4 cases per 100000 inhabitants) and the highest in 2017 (58.8 cases per 100000 inhabitants). In Cacoal, the incidence was low between August and October, whereas the incidence was high between January and April (Figure 1). The overall monthly incidence in Cacoal was 2.55 (95% CrI: 2.20-2.92) cases per 100000 inhabitants. Ariquemes, the third municipality studied, reported the highest incidence in 2007 (53.4 cases per 100000 inhabitants) and the lowest in 2008 (30.7 cases per 100000 inhabitants). A seasonal pattern was also observed for this city, where the incidence was lowest between July and September and highest between December and April (Figure 1). Ariquemes showed the highest overall monthly incidence with 3.40 (95% CrI: 3.37-3.74) cases per 100000 inhabitants. Vilhena was the city with the lowest overall monthly incidence with 2.21 (95% CrI: 1.92-2.50) cases per 100000 inhabitants. Snakebite incidence reached its lowest level in July and the highest level in March (Figure 1). The highest incidence in this city occurred during 2011 and the lowest occurred during 2012, with 36.0 and 18.8 cases per 100000 inhabitants, respectively. 

The four studied cities showed a clear seasonal pattern for rainfall, humidity, and temperature (Figure 1). Generally, high levels of rainfall had been observed towards the beginning of each year (January to March) and low levels were observed during the middle (June to August) in all cities studied (Figure 1). High levels of humidity also occurred towards the beginning of each year (January to April) and low levels had been observed during the months of June and July (Figure 1). LSTD and LSTN showed the same pattern, with both the variables exhibiting high levels closer to the end of each year (August to October) (Figure 1). 

Considering other climate variables, Porto Velho showed the highest average rainfall (Mean:149.0 mm^3^; SD: 107.0 mm^3^) and Cacoal showed the lowest (Mean: 142.0 mm^3^; SD: 116.0 mm^3^). The highest monthly average for LSTD was found in Cacoal (Mean: 32.4 °C; SD: 2.1 °C). Vilhena was the only city where temperature dropped below 20 °C; therefore, this city presented the lowest monthly average of LSTN (Mean: 19.9 °C; SD: 1.0 °C).

### Model results

**FIGURE 2:**
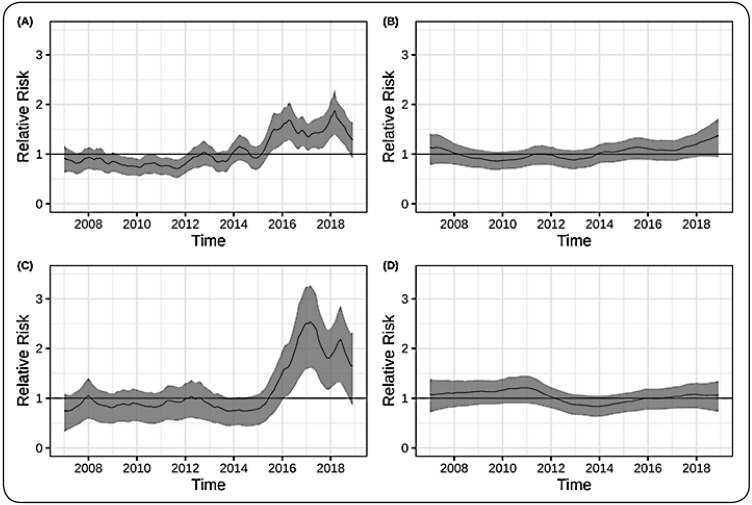
Temporal trends of the relative risk (RR) of snakebites by month from January 2007 to December 2018 in the following cities in the state of Rondonia, Brazil: **(A)** Porto Velho, **(B)** Ariquemes, **(C)** Cacoal, and **(D)** Vilhena.

The posterior mean temporal trend and 95% credible intervals by month are shown in Figure 2. These results were used to explore the temporal evolution of the relative risk (RR) of snakebites from January 2007 to December 2018 in the four cities of Rondônia, Brazil. Porto Velho and Cacoal showed a similar pattern of RR (Figure 2A and Figure 2C, respectively). However, in Porto Velho, the estimated RR was lower than that of Cacoal, and an increase in snakebite cases was initiated at the end of 2015 (Figure 2A). In the other two cities (Ariquemes and Vilhena), we did not observe any changes in the RR of snakebite accidents over time (Figure 2B and Figure 2D, respectively). Our model illustrated an increase in the RR of snakebite accidents over the last two years of the study (2016-2018) in Cacoal city, reaching a peak at the beginning of 2017 (Figure 2C).

According to the obtained data, rainfall, LSTD, and LSTN did not increase the risk of snakebite accidents in any city. However, changes in humidity seemed to increase the risk of snakebite cases for the four cities analyzed. The minimum effect of humidity was observed in Ariquemes and Cacoal, wherein an increase of 1 g/kg led to a mean increase in the risk of snakebites by 2%. The highest impact of humidity was observed in Vilhena, wherein an increase of 1 g/kg led to a mean increase in the risk of snakebites by 24% (Table 1).

**TABLE 1:** Parameter estimates for climate variables and 95% credible interval in four cities of Rondônia, Brazil, from January 2007 to December 2018.

	Porto Velho	Ariquemes	Cacoal	Vilhena
	Estimate (95% CrI)	Estimate (95% CrI)	Estimate (95% CrI)	Estimate (95% CrI)
Rainfall	1.00 (0.99-1.00)	1.00 (0.99-1.00)	0.99 (0.99-1.00)	0.99 (0.99-1.00)
Humidity	1.08 (1.03-1.14)	1.10 (1.02-1.20)	1.11 (1.02-1.22)	1.13 (1.03-1.24)
LSTD*	0.93 (0.88-0.97)	0.96 (0.91-1.02)	0.96 (0.89-0.96)	0.95 (0.85-1.05)
LSTN**	0.96 (0.89-1.03)	1.02 (1.00-1.03)	0.99 (0.97-0.99)	1.03 (0.89-1.03)
β_0_	1.35 (1.34-3.40)	-1.06 (-1.05 -1.57)	0.6 (-3.90-2.66)	1.20 (4.45-2.05)
τ_ω_	256.37 (48.25-730.16)	1194.30 (106.64-4707.33)	115.76 (26.60-344.40)	1245.80 (112.70-5023.32)
τ_δ_	60.90 (1.71-176.92)	725.69 (6.83-3522.44)	935.89 (21.89-527.90)	881.51 (17.92-3898.52)

*Land surface temperature during day; **Land surface temperature during night.

## DISCUSSION

According to SINAN, snakebite accidents were one of the most important public health problems from January 2007 to December 2018. More than 1.5 million cases occurred in Brazil. About 15000 cases of snakebites were reported from the northern region annually, of which 11358 occurs and Rondônia was the state with the fourth highest number of cases (n=11358). The four cities studied accounted for approximately 42% of the snakebites caused by a venomous species^7^. 

In this study *Bothrops* snakes were responsible for 81.80%, followed by *Laquesis* sp. (3.1%), *Micrurus* sp. (1.30%), and *Crotalus* sp. (1.21%). *Bothrops* is the most diverse genus of snakes; in this group, there are almost 30 species that can bite humans^6^. Furthermore, the species in this genus can be found in diverse environments, including cultivated areas, rural areas, and the outskirts of large cities^17^. 

Snakebite accidents caused by *Bothrops* are also common in other regions of Brazil. In Amazonas, at least 70.00% of the bites are caused by one species belonging to this genus, whereas in Amapá, this level reaches 67.5%^18^. In the present study, 79.1% of the victims were men. This observation is consistent with that reported in other studies conducted in the following states: Amazonas, Amapá, Minas Gerais, and Ceará. These studies showed that more than half of the reported cases pertained to men, resulting in an average of 73.1% of snakebite cases occurring in men^19^ ^,^ ^20^. This high percentage of male victims depicts that these incidents are related to occupational activities, such as agriculture and livestock, where male workers constitute the largest proportion of the work force.

Another relevant aspect found in the present study was that 84.5% of the snakebites occurred on the lower limbs. For instance, 50.6% of cases occurred on the feet. This percentage is consistent with that reported in other epidemiological studies that also found a prominent involvement with the lower limbs^20^.

Regarding the severity of the snakebites, the mild cases accounted for 37.4%, while the moderate and severe cases constituted 41.50% and 18% of snakebites, respectively. It was also observed that <1% (n=2) of cases resulted in death. A study conducted in the Brazilian Amazon from 2007 to 2012 found that 45.8% of the cases were moderate; however, the authors reported one death (0.6%) due to a snakebite^21^. Mortality in the present study and in the one conducted by Feitosa et al.^21^ was similar to that reported by Chippaux^22^, who stated that the mortality from snakebites is close to 0.5%. This low rate may be attributable to the efficient response to snakebites-the haste of healthcare attention and the high efficacy of the serum therapy.

In this study, a clear seasonal variation in snakebite incidence was observed. The pattern seemed to be similar among the cities, with higher incidences at the beginning of the year, reaching the peak in March; meanwhile, the incidences decreased, starting in July, reaching their lowest in August. The seasonal patterns of the snakebite incidences described here are consistent with that reported by Feitosa et al.^21^, who conducted a systematic literature review in the Amazon region. Machado et al.^23^, using data from SINAN, also observed a high incidence of snakebite accidents in March and low incidence of snakebite accidents in August. The climate variables were mostly used to explain the seasonality of snakebite accidents. Machado et al.^23^ reported that higher incidences of snakebites occurred during the months with the highest temperatures. However, Philips et al.^24^ found that higher incidences of snakebites were inversely correlated with drought. Roriz et al.^9^have reported that about 78.3% of snakebite accidents occur between November and April; this corresponds to the Amazon’s rainy season. A study conducted in India in 2013 reported that accidents and deaths caused by snakebites were more during the years with higher levels of rainfall^25^. 

In the present study, we assessed the relationship between four climate variables (rainfall, humidity, LSTD, and LSTN) and the incidence of snakebite accidents. Of the variables analyzed, only humidity seemed to be related to the incidence of snakebites. We found that, depending on the city, an increase of 1 g/kg in humidity could lead to a mean increase in the risk of snakebite cases by 24%. The relationship between humidity and the increased risk of snakebites is owing to the fact that in humid seasons, the species that snakes feed on (such as anurans, lizards, and small mammals) are more active; further, their young are born at this time^26^ ^,^ ^27^ ^,^ ^28^. At the same time, *Bothrops* are also more active, and their pups are born^29^ ^,^ ^30^ ^,^ ^31^ ^,^ ^32^ ^,^ ^33^ ^,^ ^34^ ^,^ ^35^. Coincidentally, there is an intensification of human activities in rural areas, favoring the encounter between humans and snakes^36^ ^,^ ^37^ ^,^ ^38^.

## CONCLUSIONS

The current study depicts the scenario of snakebites occurring in the Rondônia state between the years of 2007 and 2018. It was observed that these cases are a serious public health problem in this state, especially in the micro region of Porto Velho. This study also identified the population exposed to the potential of snakebites, as well as the influence of anthropic and climatic factors. This study found that the majority of the reported cases occurred in men, primarily engaging in field activities, such as agriculture or livestock. These cases occurred more commonly during rainy periods, with relatively higher humidity and milder temperatures, with the genus *Bothrops* responsible for about 87% of the snakebite cases. In addition, it was also observed that the lower limbs were the most affected. According to climate data, changes in humidity increased the risk of snakebite accidents for the four cities analyzed; however, Porto Velho and Cacoal had higher snakebite RRs than Ariquemes and Vilhena.
